# A Glutamine-Rich Factor Affects Stem Cell Genesis in Leech

**DOI:** 10.4061/2010/145183

**Published:** 2009-11-15

**Authors:** Kristi A. Hohenstein, Shirley A. Lang, Tej Nuthulaganti, Daniel H. Shain

**Affiliations:** Department of Biology, Rutgers, The State University of New Jersey, Camden, NJ 08102, USA

## Abstract

Leech embryogenesis is a model for investigating cellular and molecular processes of development. Due to the unusually large size of embryonic stem cells (teloblasts: 50–300 *μ*m) in the glossiphoniid leech, *Theromyzon tessulatum*, and the presence of identifiable stem cell precursors (proteloblasts), we previously isolated a group of genes upregulated upon stem cell birth. In the current study, we show that one of these genes, designated *Theromyzon* proliferation (*Tpr*), is required for normal stem cell genesis; specifically, transient *Tpr* knockdown experiments conducted with antisense oligonucleotides and monitored by semiquantitative RT-PCR, caused abnormal proteloblast proliferation leading to embryonic death, but did not overtly affect neuroectodermal or mesodermal stem cell development once these cells were born. *Tpr* encodes a large glutamine-rich (∼34%) domain that shares compositional similarity with strong transcriptional enhancers many of which have been linked with trinucleotide repeat disorders (e.g., Huntington's).

## 1. Introduction

Stem cells (SCs) are unique in that they self-renew and generate differentiated cell types; knowledge of the genes that govern these processes is clearly important in determining their genetic potential. Various transcriptional profiling efforts have identified candidate genes involved in SC self-renewal and potency [1–3], but little overlap occurs between gene datasets, which has called these analyses into question [4]. Nonetheless, a few genes are generally linked with stem cell genesis or maintenance (e.g., Oct4 [5]; Nanog [6]; Sox2 [7]), and certain combinations of transcription factors appear sufficient to promote stem cell fate when ectopically expressed in some nonstem cell types [8, 9].

**Figure 1:**
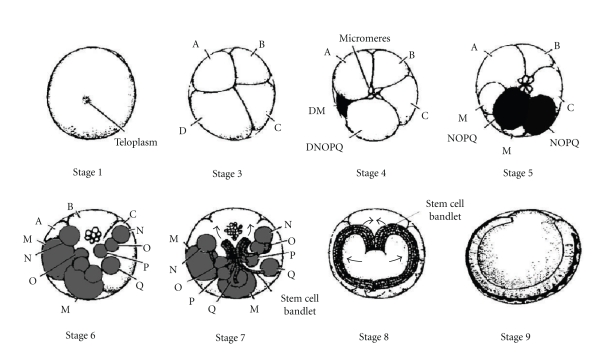
Schematic of early development in glossiphoniid leeches. After several asymmetric cleavages, proteloblasts DM (stage 4) and NOPQ (stage 5) are stereotypically positioned on the embryo's surface. A series of unequal divisions leads to five bilaterally paired stem cells (10 total), or teloblasts (gray cells: M, N, O, P, Q), that give rise to chains of segmental founder cells called bandlets (stages 7, 8). By epiboly, bandlets move across the surface of the embryo (arrows, stage 8) and coalesce to form the segmental mesodermal and ectodermal tissue. Modified from Hohenstein and Shain [10].

Current stem cell research has focused on mammalian SCs, but we have explored this topic in an invertebrate model system, the leech *Theromyzon tessulatum*, which transiently displays embryonic SCs that have properties similar to mammalian adult SCs [10]. Leech offers a new perspective to this arena because stem cell precursors (proteloblasts) and stem cells (teloblasts) are experimentally accessible during development and, in contrast to mammalian embryonic cells, homogeneous populations of both leech cell types can be prepared by relatively simple dissections (Figure 1). Leech proteloblasts and teloblasts are particularly large (50–400 *μ*m), which permits cell type-specific microinjections of molecular reagents (e.g., lineage tracers, nucleic acids), and they appear at predictable spatiotemporal positions during embryogenesis (Figure 1). Specifically, the mesodermal proteloblast (DM) and ectodermal proteloblasts (NOPQ left and right) are derived from the D quadrant macromere within ~10 hours after fertilization and persist for an ~1 hour time window; thereafter, each proteloblast gives rise to its respective teloblast(s) lineage at the rate of ~1 cleavage per hour (i.e., DM cleavage generates left and right M teloblasts, and NOPQ gives rise to N, OP, Q, O, and P teloblasts in successive cleavages). 

Taking advantage of these developmental features, we previously used differential display-PCR (DD-PCR) methodology to identify a set of unbiased (i.e., noncandidate gene approach), differentially expressed genes, several of which are upregulated upon the birth of teloblasts [10]. Following up on our previous work, we report here that one teloblast-specific gene, designated *Theromyzon* proliferation (*Tpr*), encodes a glutamine-rich protein that plays a critical role in teloblast formation. Specifically, *Tpr* is up-regulated during the conversion of proteloblasts to teloblasts, and *Tpr* knockdown experiments disrupt normal cell cleavage patterns resulting in the abnormal proliferation of targeted proteloblast, but not teloblast, cells.

## 2. Materials and Methods

### 2.1. Leeches and Embryo Collection

Adult *Theromyzon tessulatum *specimens, formerly confused with *T. rude* or *T. trizonare *[11], were collected in the ponds of Golden Gate Park (San Francisco, CA). Leeches were maintained at 12°C in 0.03% Instant Ocean Salt (Aquarium Systems). Embryos were staged by visual inspection under a stereomicroscope, and appropriate stages were harvested as described [10].

### 2.2. Differential Display-PCR

Microdissection and collection of individual cells from leech embryos and their transcriptional profiling by differential display-PCR (DD-PCR) were conducted as described in [10]. DD-PCR bands were amplified according to the manufacturer's protocol (Clontech); bands were gel purified with a Minielute Gel Extraction kit (Qiagen) and cloned into pGEM-T Easy (Promega). DNA was sequenced commercially (Northwoods DNA, Inc., Becida, MN) with standard T7 or Sp6 primers.

### 2.3. cDNA Library Screening and RACE (Rapid Amplification of cDNA Ends)

*λ* Triplex2 cDNA libraries were constructed from ~100 stage 1 *T. tessulatum* embryos (maternal library) and an assortment of stages 1–9 embryos (embryonic library) using SMART methodology (Clontech). Library screening was conducted under high stringency by standard procedures [12], with [^32^P]-dCTP PCR-labeled probes. RACE-PCR was conducted using *Tpr*-specific oligonucleotides (TprA-D; see below) derived from the original differentially expressed *Tpr* fragment [10].

### 2.4. Oligonucleotide Microinjections

Oligonucleotides were synthesized commercially (Sigma-Genosys). Antisense oligonucleotides were TprA—TTGATATTACTGCCAGCATG, TprB—TGTAGTTGTCGTTGATGTTG; sense oligonucleotides were TprC—TGCAACACATTCGACATCAC, TprD—AACACACGACAACAACAACG. Oligonucleotides were resuspended in H_2_O at 1 mM and coinjected with fluorescent lineage tracer (either fluorescein-dextran amine (FDA, Molecular Probes) or tetramethylrhodamine-dextran amine (RDA, Molecular Probes)) as described [13].

### 2.5. Imaging

Embryos were viewed on a Zeiss Axioplan equipped with epifluorescence. Images were captured with a Nikon Coolpix 5000 camera and processed in Photoshop (Adobe).

### 2.6. Semiquantitative Reverse Transcription—Polymerase Chain Reaction (RT-PCR)

Total RNA was extracted using the Total RNA Isolation System (Promega) and reverse transcribed with Powerscript (Clonetech). First-strand cDNA was amplified using commercially synthesized (Sigma-Genosys), gene-specific primers and 18S ribosomal RNA primers. RT-PCR primer sets are listed below with approximate fragment size: Tpr1—TTGTCAAAACAACGTGACAAC, Tpr2—GGTTTTTGTTGTTGAATGCTG (270 bp); 18S ribosomal RNA—GCTTGTCTCAAAGATTAAGCC, AACTACGAGCTTTTTAACTGC (610 bp). RT-PCR was conducted with Titanium Taq DNA polymerase (Clontech) using the following parameters: 94°C (15 seconds); 57°C (1 minute); 72°C (1 minute) for 32 cycles. Each presented lane is representative of at least three independent experiments.

## 3. Results

**Figure 2:**
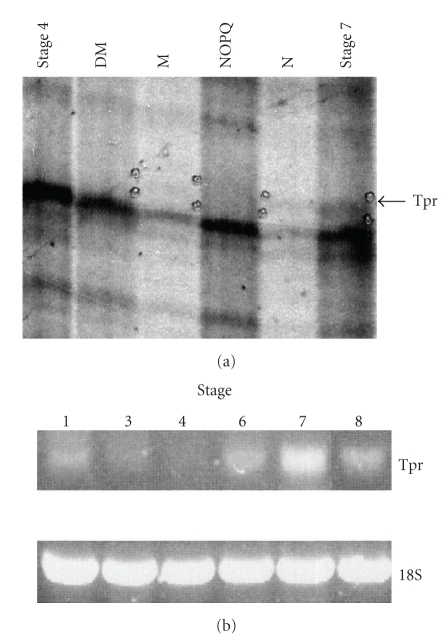
Differential expression of *Tpr* during leech embryogenesis. (a) Differential display analysis shows faint *Tpr* bands (boxed with pinholes) in teloblast lanes M and N, and in stage 7 embryos which contain teloblasts M, N, O, P and Q. Bands above and below appear in all lanes and are likely “housekeeping” genes. (b) Semi-quantitative RT-PCR analysis shows declining *Tpr* transcripts during stage 1 (presumably maternal) through stage 4; accumulation of new zygotic transcripts was evident thereafter and peaked at stage 7. Lanes were normalized with 18S rRNA primers.

Among several genes upregulated upon the birth of leech teloblasts, *Tpr* displayed an unambiguous, albeit weak, teloblast-specific expression profile in differential display (DD) analysis (i.e., bands in M and N teloblast lanes and also stage 7 embryos; see Figure 2(a)). Relatively weak DD bands were consistent with negative Northern blots using a *Tpr *probe, suggesting that *Tpr *is likely a rare transcript. To verify the DD pattern of *Tpr *and further analyze its temporal expression during leech embryogenesis, semiquantitative reverse transcriptase-PCR (RT-PCR) was conducted using appropriately staged *Theromyzon tessulatum* embryos. Embryos at stages 1–4 (containing proteloblast cells), stage 6 (containing all 10 teloblasts), and stages 7 and 8 (containing teloblasts and bandlets—see Figure 1) were collected and processed for RT-PCR. To normalize reactions, 18S ribosomal RNA was coamplified in all reactions. These analyses demonstrated that *Tpr* was upregulated in embryos containing teloblasts, in comparison with their immediate precursors, DM, and NOPQ (Figure 2(b)). Surprisingly, *Tpr* mRNA was also detected in the fertilized egg (stage 1), indicating its presence as a maternal mRNA. Transcript levels declined during early cleavages until reaching near background levels in stage 4 embryos, which contain proteloblast cells, DM and NOPQ. Comparable declines of maternal transcripts have been observed in leech (e.g., *nanos *[14]) and other organisms (e.g., mouse [15]). Coincident with the birth of teloblasts, *Tpr* levels increased until reaching peak levels at stage 7 (containing teloblasts and bandlets) before declining by stage 8.

**Figure 3:**
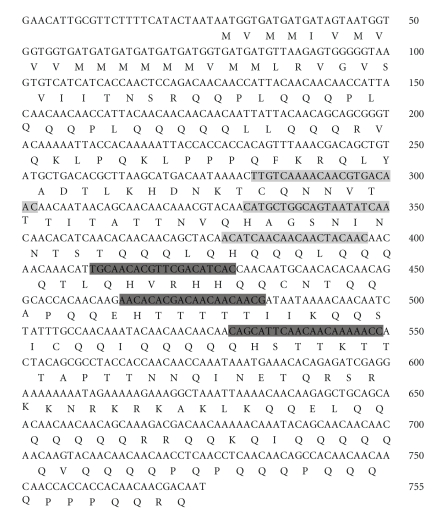
*Tpr* nucleotide and predicted amino acid sequences (GenBank accession no. EU527977). The available ORF encodes a protein domain that contains ~34% glutamine. Shaded sequences identify the positions of oligonucleotides TprA (330–350), TprB (378–397), TprC (410–429), TprD (464–483), Tpr1 (292–302), and Tpr2 (529–549) that were employed for microinjections, RACE-PCR and RT-PCR (see Section 2).

Because DD analysis generates only gene fragments, *T. tessulatum *cDNA libraries were screened in efforts to obtain additional *Tpr* cDNA sequences. Overlapping cDNA fragments obtained from multiple library screens and RACE-PCR products generated a combined linear sequence of 775 bp that includes a glutamine-rich (~34%) open reading frame of 249 amino acids (Figure 3). Curiously, the 3′ end of *Tpr* appears unrepresented in our oligo dT-primed maternal and embryonic cDNA libraries, suggesting an internal A-rich segment (note the abundance of CAA and CAG repeats in the available sequence), and we were unable to obtain additional 3′ sequence by RACE-PCR using staged 1st strand cDNA as template. Likewise, 3′ sequences beyond that shown in Figure 3 were not detected in our available *T. tessulatum* genomic library.

**Figure 4:**
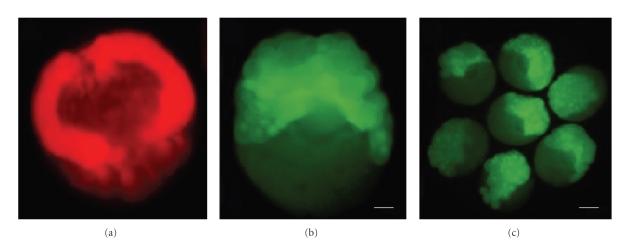
Proliferated cell clusters generated after microinjecting *Tpr* antisense oligonucleotides into developing *Theromyzon tessulatum* embryos. (a) Normal, mid-stage 8 embryo fixed ~48 hours after coinjecting sense oligonucleotide TprC and RDA (red) into the D macromere. (b) Representative embryo fixed ~48 hours after co-injecting antisense oligonucleotide TprB and FDA (green) into the D macromere. (c) Sampling of embryos from the same experimental group as in (b). Scale bar = 100 *μ*m (a), (b); 250 *μ*m (c).

To examine the function of *Tpr *in developing embryos, an assortment of antisense (AS) oligonucleotides was generated to transiently knockdown *Tpr* mRNA levels during embryogenesis. To control for specificity and toxicity, independent sense oligonucleotides were microinjected into developing embryos at various stages, none of which caused overt embryonic defects (Figure 4(a)). However, microinjection of three independent *Tpr* AS oligonucleotides into the D macromere of stage 3 embryos (see Figure 1) caused abnormal cell divisions of DM and NOPQ cells, leading to disorganized cell clusters (representative phenotypes shown in Figures 4(b) and 4(c)). Note that AS oligonucleotides targeting other teloblast-specific genes identified in our original DD-PCR analysis displayed fundamentally different phenotypes (e.g., bandlet truncation), none of which were related to the abnormal proliferation resulting from *Tpr* AS oligonucleotides [16]. In total, we observed this clustered cell phenotype in >300 experimental embryos from >5 independent clutches, with ~95% of embryos affected; the remaining embryos were typically overinjected and did not divide further following the initial microinjection. The efficacy of independent AS oligonucleotides varied (consistent with other reports [17]), with TprB (both phosphodiester and phosphorothioate linked) displaying a stronger phenotype than others tested; consequently, AS oligonucleotide TprB was employed for subsequent analyses. Note that disorganized cells could not be counted accurately since they formed three-dimensional arrays; however, our estimates suggest that the total number of cells in TprB-injected embryos approximated the normal number of cells in comparably staged embryos, if micromeres were factored into the counts (compare also control versus experimental embryos in Figure 6). About 48 hours postinjection, cell proliferation ceased and embryos died shortly thereafter. Typically, several hundred abnormal cells (roughly equal in size; see Figures 4(b) and 4(c)) were derived from one antisense-injected D macromere.

**Figure 5:**
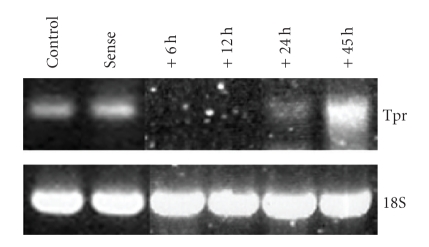
Transient knockdown of *Tpr* mRNA as monitored by semi-quantitative PCR. Antisense oligonucleotide TprB was microinjected into the D macromere of ~50 embryos, and ~12 embryos were harvested 6 (stage 6), 12 (stage 7), 24 (early stage 8) and 45 (mid-stage 8) hours later, respectively. *Tpr* mRNA levels were apparent by 24 hours post-injection in proliferated embryos. Control (no injection) and sense (TprC) microinjections were conducted in the same experimental clutch and harvested at 6 (stage 6) and 12 (stage 7) hours post-injection, respectively. Lanes were normalized with 18S rRNA primers.

Semi-quantitative RT-PCR analyses demonstrated that *Tpr* mRNA levels were knocked down in the experiments described above (Figure 5). Specifically, *Tpr* transcripts were not detected in AS-injected embryos undergoing abnormal proliferation at 6 hours (stage 6) or 12 hours (stage 7) post-injection, yet were readily detected in corresponding uninjected and sense-injected control embryos, respectively (Figure 5). By ~24 hours post-injection (early stage 8 in normal embryos) *Tpr* transcripts appeared in AS-injected embryos, and increased further at ~45 hours (mid-stage 8 in normal embryos), just prior to embryonic lethality.

**Figure 6:**
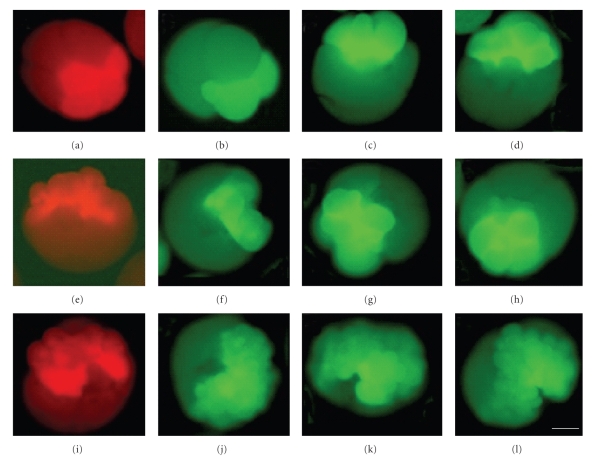
Time course of cell divisions leading to proliferated cell clusters following *Tpr* antisense oligonucleotide microinjections into the D macromere. (a) Normal embryo ~6 hours after microinjection with sense oligonucleotide TprC (co-injected with RDA; red). (b)–(d) Representative atypical cleavages ~6 hours after microinjection with antisense oligonucleotide TprB (co-injected with FDA; green). (e) Normal embryo ~12 hours post-injection. (f)–(h) Antisense injected embryos ~12 hours post-injection. (i) Normal embryo ~24 hours post-injection. (j)–(k) Antisense injected embryos ~24 hours post-injection. Scale  bar = 200 *μ*m.

**Figure 7:**
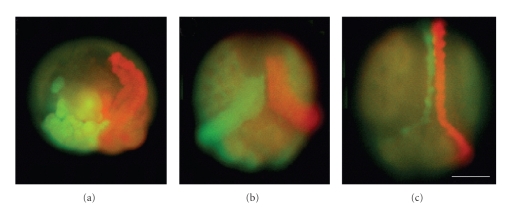
*Tpr* antisense oligonucleotides affected proteloblast, but not teloblast, cell development. (a) Proteloblast NOPQ (right) was microinjected with *Tpr* sense oligonucleotide TprC (co-injected with RDA; red) and displayed a normal bandlet pattern, while NOPQ (left) microinjected with *Tpr* antisense oligonucleotide TprB (co-injected with FDA; green) proliferated abnormally; representative embryo harvested at ~36 hours post-injection. (b) M (right) teloblast microinjected with TprC (sense; red) appeared normal ~50 hours later; M (left) microinjected with TprB (antisense; green) also appeared normal (the disparity in the extent of each germinal band resulted from the ~2 hours delay between sense and antisense microinjections). (f) N (right) teloblast microinjected with TprC (sense; red) appeared normal ~72 hours later; N (left) microinjected with TprB (antisense; green) appeared relatively normal. Scale bar = 200 *μ*m.

To determine the time window in which *Tpr* expression was critical for normal development, different cell types were microinjected with AS and sense oligonucleotides and monitored for abnormal proliferation (Figures 6 and 7). Embryos injected with AS oligonucleotides at the 1-2 cell stage developed normally until stage 4, at which point the mesodermal proteloblast (DM), which normally buds off two micromeres before dividing equally into M_L_ and M_R_ teloblasts (Figure 6(a)), divided off-center (and unpredictably) in comparison with its normal cleavage pattern (Figures 6(b)–6(d)). Thereafter, cell cleavages in AS-injected cells were irregular and displayed no detectable patterns, but typically generated abnormal cell clusters with roughly equal-sized cells (Figures 6(e)–6(l); cf.  Figure 4). Similarly, knockdown of *Tpr* mRNA in proteloblasts DM and NOPQ caused abnormal cleavages in mesodermal (M) and neuroectodermal (N, O, P, and Q) lineages, respectively (e.g., Figure 7(a)), and also abnormal cell divisions, but to a lesser extent than observed in earlier-staged AS injections. In general, the extent of proliferation in each embryo was directly related to the age of the injected cell; thus, early embryonic injections (i.e., stages 1, 2, and 3) resulted in larger cell clusters, while later injections (i.e., precursors DM and DNOPQ) were less severe (Figure 7(a)). 

Finally, *Tpr* antisense injections into M, N, and OP teloblasts did not induce abnormal proliferation, but rather had little or no effect on subsequent development (e.g., bandlet formation) in comparison with contralateral, sense-injected or noninjected teloblast lineages (Figures 7(b) and 7(c)). Note that these injections were conducted just a few hours later than AS injections into DM (which caused dramatic anomalies), and levels of *Tpr* mRNA were likely comparable at the different experimental stages (see Figure 2(b)). Thus, a short developmental time window existed (i.e., a few hours preceding teloblast birth) within which *Tpr* mRNA knockdown experiments prevented normal teloblast genesis, suggesting that inhibiting the onset of presumptive zygotic *Tpr* transcription (cf.Figure 2(b)) was more crucial for normal teloblast development than knocking down *Tpr* transcripts once teloblasts were born.

## 4. Discussion

By comparing gene expression profiles in proteloblast and teloblast cells, we isolated a relatively small set of teloblast-specific genes [10], one of which encodes the glutamine-rich factor designated *Tpr*. The defined developmental time window within which *Tpr* knockdown experiments were effective (i.e., a few hours prior to the birth of teloblasts) suggests that *Tpr* AS oligonucleotides prevented the normal onset of presumptive *Tpr* transcripts, implying that Tpr acts primarily at the proteloblast → teloblast transition point, and not in the maintenance of teloblasts once differentiated. Note, however, that Tpr must act prior to proteloblast cleavage (i.e., in the proteloblast), since *Tpr* knockdowns clearly affected the proteloblast cleavage pattern (see Figure 6). Considering the spatiotemporal expression of *Tpr* presented here (i.e., DD-PCR, RT-PCR), we propose that *Tpr* is a rare transcript expressed shortly before proteloblast cleavage and is required for normal teloblast birth. 

This time window corresponds roughly with the onset of full-blown zygotic transcription in the related leech, *Helobdella robusta*, though zygotic transcription has been detected at the 2-cell stage [18]. Full transition to zygotic control in leech is similar to models like sea urchin, where zygotic transcription begins during early cleavages but full zygotic control is not present until later in development [19]. When transcription in *H. robusta* was inhibited by *α*-amanitin, aberrant cell cleavages resulted just prior to teloblast formation leading to embryonic death [19], comparable to our 6–12 hours postinjection phenotypes (see Figure 6) but differing in the extent of abnormal cell divisions and time frame of lethality. Nonetheless, parallels between the two studies are evident, and differences may be related to the number of genes targeted in each study and/or species-specific developmental variation; indeed, morphologically indistinguishable species of *Helobdella* not only diverge significantly in their genome sequence, but also display notable dissimilarities in developmental processes [20].

### 4.1. Cell Proliferation

The extent to which embryonic cells proliferated following *Tpr* AS injections into proteloblast cells has not been previously observed in leech, but the propensity of embryonic cells to proliferate abnormally has been documented by the misexpression of several genes, particularly in the fruit fly, *Drosophila melanogaster.* For example, a *lethal giant larvae *knockout causes abnormal proliferation of neuroblasts in epithelia [21], mutations in *Rpb9 *that prevent its expression in stage 3 egg chambers cause cystocytes to overproliferate leading to an ovarian tumor phenotype [22], and loss of notch signaling in the central nervous system causes extensive cell proliferation [23]. Although we are currently unable to establish the mechanism by which *Tpr* downregulation causes abnormal cell proliferation, our data link zygotic *Tpr* expression with the normal birth of leech teloblasts; when zygotic *Tpr* expression is inhibited, proteloblast cells displayed irregular cleavages that led to abnormal cell masses and embryonic death. In principle, the *Tpr* gene product may be associated with the orientation of the proteloblast cleavage plane; loss of this control may disrupt normal partitioning, cell-cell interactions and/or signaling leading to the constitutive division of daughter cells (cf. *lethal giant nerve, Rpb9,* and *notch* knockouts described previously).

### 4.2. Glutamine-Rich Proteins

Glutamine-rich domains have been linked with protein-protein interactions (e.g., polar zippers) that influence transcription. For instance, the strong enhancer protein Sp1 interacts with components of TFIID through a glutamine-rich domain [24], and notch signaling is mediated by LAG3, a glutamine-rich transcription factor that lacks a DNA binding motif [25]. The propensity of CAG trinucleotide repeats to expand during gametogenesis (leading to stretches of glutamine repeats) is the fundamental cause of Huntington's and related diseases [26, 27] and contributes to the lack of sequence similarity observed between glutamine-rich homologues of related taxa [28]. 

We were nonetheless surprised to find only compositional matches (i.e., glutamine-rich domains) to *Tpr* in GenBank; on the other hand, transcription factors with homopolymeric runs of specific amino acids (i.e., Gln, Ser, Ala, Pro, Gly) that are components of well-conserved *Hox* gene regulatory pathways (e.g., *lag-3 *and *sop-3 *in *C. elegans*; *mastermind *in *D. melanogaster*) do not have clear homologues in other organisms [28]. Perhaps *Tpr* has evolved rapidly in the *Theromyzon* lineage (a derived group of oligochaetes [29]), or gaps may exist in the genome sequences of other leeches, possibly related to our difficulties in cloning the *Tpr *3′ end. Regardless, Tpr seems likely to function at a transcriptional level based on its hierarchical mRNA expression in newborn teloblasts, and its characteristic glutamine-rich domain that is a hallmark of transcriptional activation domains [27, 30].

## 5. Conclusion

We report a glutamine rich factor (*Tpr*) that is up-regulated upon stem cell genesis in leech. Genetic knockdowns of *Tpr* in early stage embryos prevent normal stem cell genesis, resulting in abnormal cell proliferation and embryonic death; Tpr knockdowns following stem cell birth display no overt developmental abnormalities.
